# Supplementary material for: t‐Darpp is an elongated monomer that binds calcium and is phosphorylated by cyclin‐dependent kinases 1 and 5
Source: FEBS Open Bio. 2017 Aug 29;7(9):1328–37. doi: 10.1002/2211-5463.12269 (PMC5586343; doi:10.1002/2211-5463.12269)
Supplement: Supplementary file 1 — Fig. S1. Circular dichroism of t‐Darpp. Fig. S2. Circular dichroism of Darpp‐32. Fig. S3. Circular dichroism absorbance spectrum of t‐Darpp upon addition of metal ions. Fig. S4. Circular dichroism absorbance spectrum of Darpp‐32 upon addition of metal ions. Table S1. Graphite furnace parameters for calcium level measurements. Calculation S1. Classification of a t‐Darpp as a highly elongated protein 18. [file FEB4-7-1328-s001.pdf]

SUPPLEMENTARY MATERIALS

Figure S1. Circular dichroism of t-Darpp.

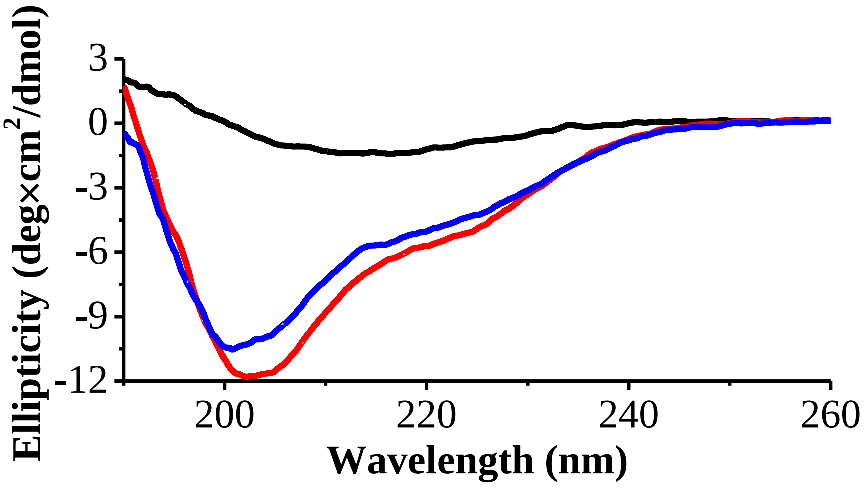

Figure S1. Circular dichroism of t-Darpp. Black, buffer; red, 0.12 mg/mL t-Darpp at ambient temperature; blue, 0.12 mg/mL t-Darpp heat treated for 5 min at 50°C and returned to ambient temperature.

Figure S2. Circular dichroism of Darpp-32.

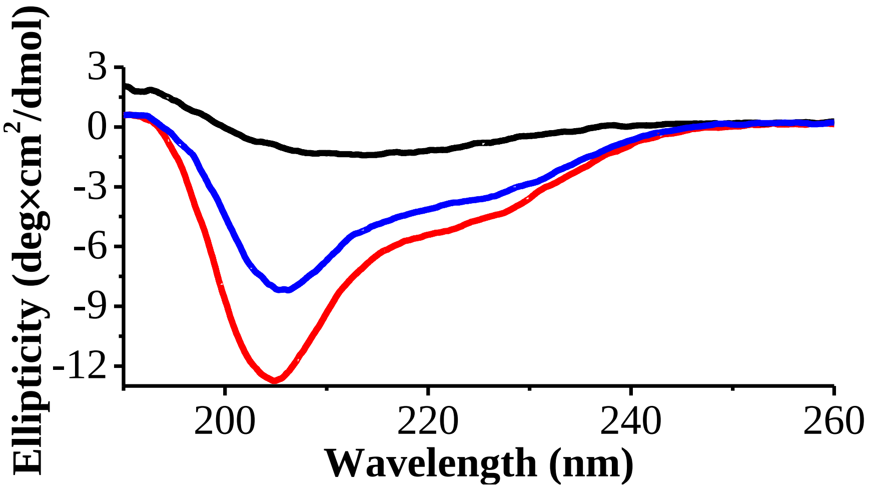

Figure S2. Circular dichroism of Darpp-32. Black, buffer; red, 0.10 mg/mL Darpp-32 at ambient temperature; blue, 0.10 mg/mL Darpp-32 heat treated for 5 min at 50°C and returned to ambient temperature.

Figure S3. Circular dichroism absorbance spectrum of t-Darpp upon addition of metal ions.

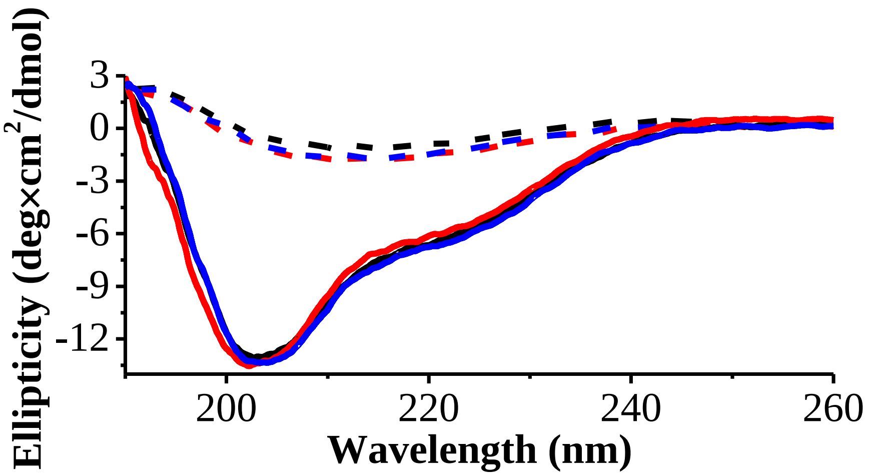

Figure S3. Circular dichroism absorbance spectrum of t-Darpp upon addition of metal ions. Black dash, buffer; blue dash, buffer + 1 mM $MgSO_4$; red dash, buffer + 1 mM $Ca(H_2PO_4)_2$; black solid, t-Darpp; blue solid, t-Darpp + 1 mM $MgSO_4$; red solid, t-Darpp + 1 mM $Ca(H_2PO_4)_2$. Experiment was conducted at ambient temperature.

Figure S4. Circular dichroism absorbance spectrum of Darpp-32 upon addition of metal ions.

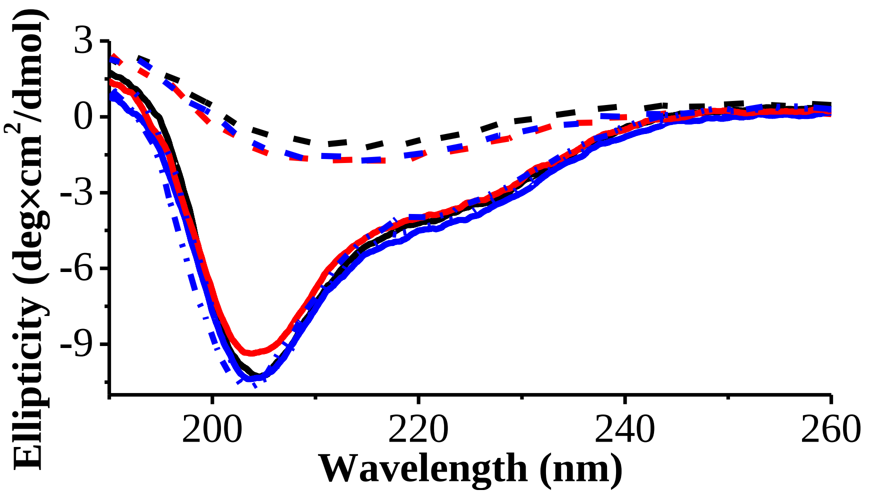

Figure S4. Circular dichroism absorbance spectrum of Darpp-32 upon addition of metal ions. Black dash, buffer; blue dash, buffer + 1 mM $MgSO_4$; red dash, buffer + 1 mM $Ca(H_2PO_4)_2$; black solid, Darpp-32; red solid, Darpp-32 + 1 mM $Ca(H_2PO_4)_2$; blue dash dot dot, Darpp-32 + 10 μM $MgSO_4$; blue dot, Darpp-32 + 100 μM $MgSO_4$; blue solid, Darpp-32 in buffer + 1 mM $MgSO_4$. Experiment conducted at ambient temperature.

Table S1. Graphite furnace parameters for calcium level measurements.

Stage	Temperature (^0C)	Time(s)	Heat mode	Ar rate (L/min)
1	150	20	Ramp	0.10
2	250	10	Ramp	0.10
3	800	10	Ramp	1.00
4	800	10	Step	1.00
5	800	3	Step	0.20
6	2200	2	Step	0.20
7	2500	2	Step	1.00

Calculation S1

Classification of a t-Darpp as a highly elongated protein [18]

Calculated mass of recombinant t-Darpp = 19,578.67 Da

R_{min} is the minimal radius of a sphere that could contain the given mass of protein, R_S is the Stokes radius, f_{min} is minimum frictional coefficient, and f is the frictional coefficient calculated from the Stokes radius.

1. Calculation of R_{min}

$R_{min} = 0.066 * M^{1/3}$ (M in Dalton, R_{min} in nanometer)

$R_{min} = 0.066 * 19578.67^{1/3} = 1.8$ nm

2. R_s of tDarpp

Stokes radius measurement of t-Darpp from Superose gel filtration chromatography = 4.4 nm

$R_s/R_{min} = 4.4 / 1.8 = 2.4$

$R_s/R_{min} = f/ f_{min} = 2.4$.

When f/f_{min} falls within the range of 2.0 to 3.0 the protein is classified as highly elongated.
